# Extragenital Lichen Sclerosus With Vulvar Involvement in a Child: A Clinical and Dermoscopic Perspective

**DOI:** 10.7759/cureus.101188

**Published:** 2026-01-09

**Authors:** Layla Bendaoud, Meryem Aboudourib, Khaoula Jaatar, Said Amal, Ouafa Hocar

**Affiliations:** 1 Dermatology and Venerology, Mohammed VI Centre Hospitalier Universitaire (CHU) Bioscience and Health Research Lab, Faculty of Medicine and Pharmacy - Cadi Ayyad University, Marrakesh, MAR; 2 Dermatology, Mohammed VI Centre Hospitalier Universitaire (CHU) Bioscience and Health Research Lab, Faculty of Medicine and Pharmacy - Cadi Ayyad University, Marrakesh, MAR

**Keywords:** children, cutaneous, extragenital involvement, lichen sclerosus, lichen sclerosus et atrophicus

## Abstract

Lichen sclerosus is a chronic inflammatory condition that involves both anogenital and extragenital areas, primarily affecting females, and can occur at any age. Although extragenital manifestations are rare, children may present with genital involvement, extragenital lesions, or a combination of both. We report a case of an eight-year-old girl who had vulvar lesions for two years, followed by the appearance of widespread cutaneous lesions two months prior to presentation. The diagnosis of lichen sclerosus was confirmed through dermoscopic evaluation and histological examination.

## Introduction

Lichen sclerosus is a chronic inflammatory disease that most commonly affects the anogenital region, although any part of the body may be involved. It shows the following two incidence peaks: one in prepubertal girls and another in adult women, with females being more frequently affected than males [1]. In the pediatric population, extragenital involvement is uncommon. In this study, we present a case of an eight-year-old girl with vulvar lichen sclerosus associated with extragenital lesions.

## Case presentation

**Figure 1:**
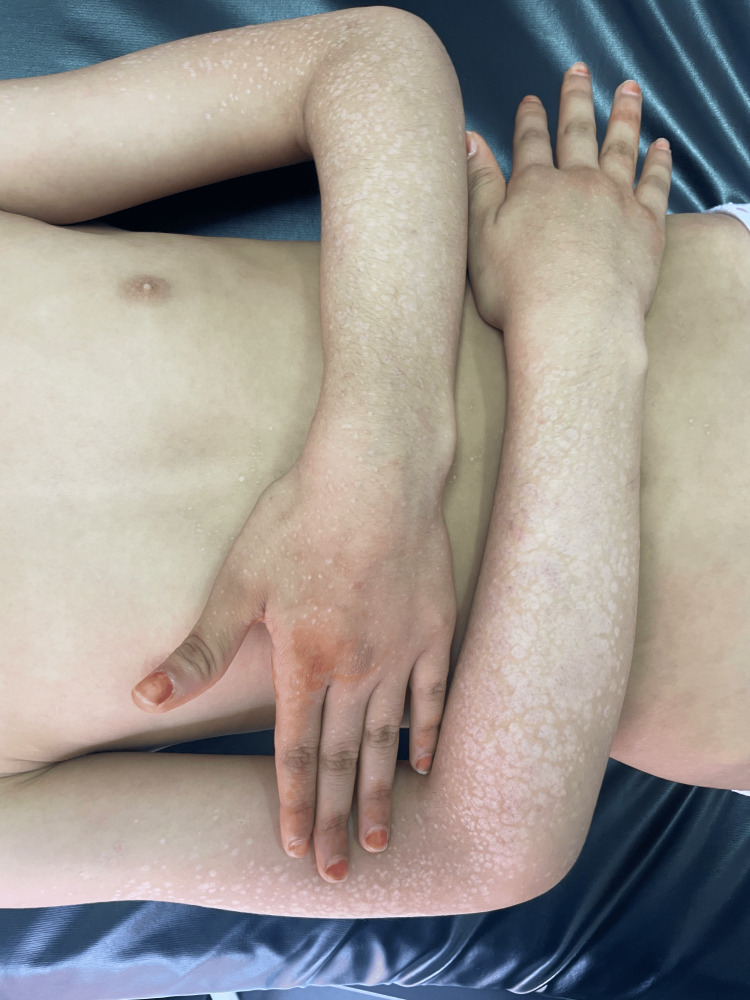
Generalized eruption with atrophic whitish lesions on the upper limbs.

**Figure 2:**
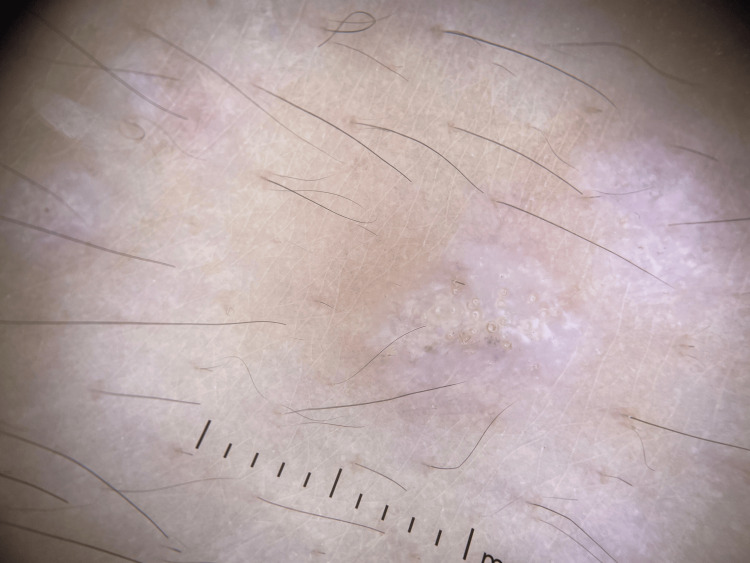
Dermoscopy of Lichen sclerosus. Dermoscopy found homogeneous whitish areas associated with yellowish circular structures, consistent with comedo-like openings and a few irregular vessels.

An eight-year-old girl presented with a two-year history of pruritic lesions in the genital area. Two months prior to evaluation, she developed white lesions on her face and extremities. Dermatological examination revealed multiple shiny white papules, some with an atrophic appearance. These lesions were distributed over the face and both upper and lower limbs, predominantly on the dorsal surfaces of the forearms and legs (Figure 1). In the genital area, an erythematous plaque with areas of hypopigmentation and superficial erosions was observed. Dermoscopy of the extragenital lesions revealed homogeneous whitish areas associated with yellowish circular structures, consistent with comedo-like openings and a few irregular vessels (Figure 2).

**Figure 3:**
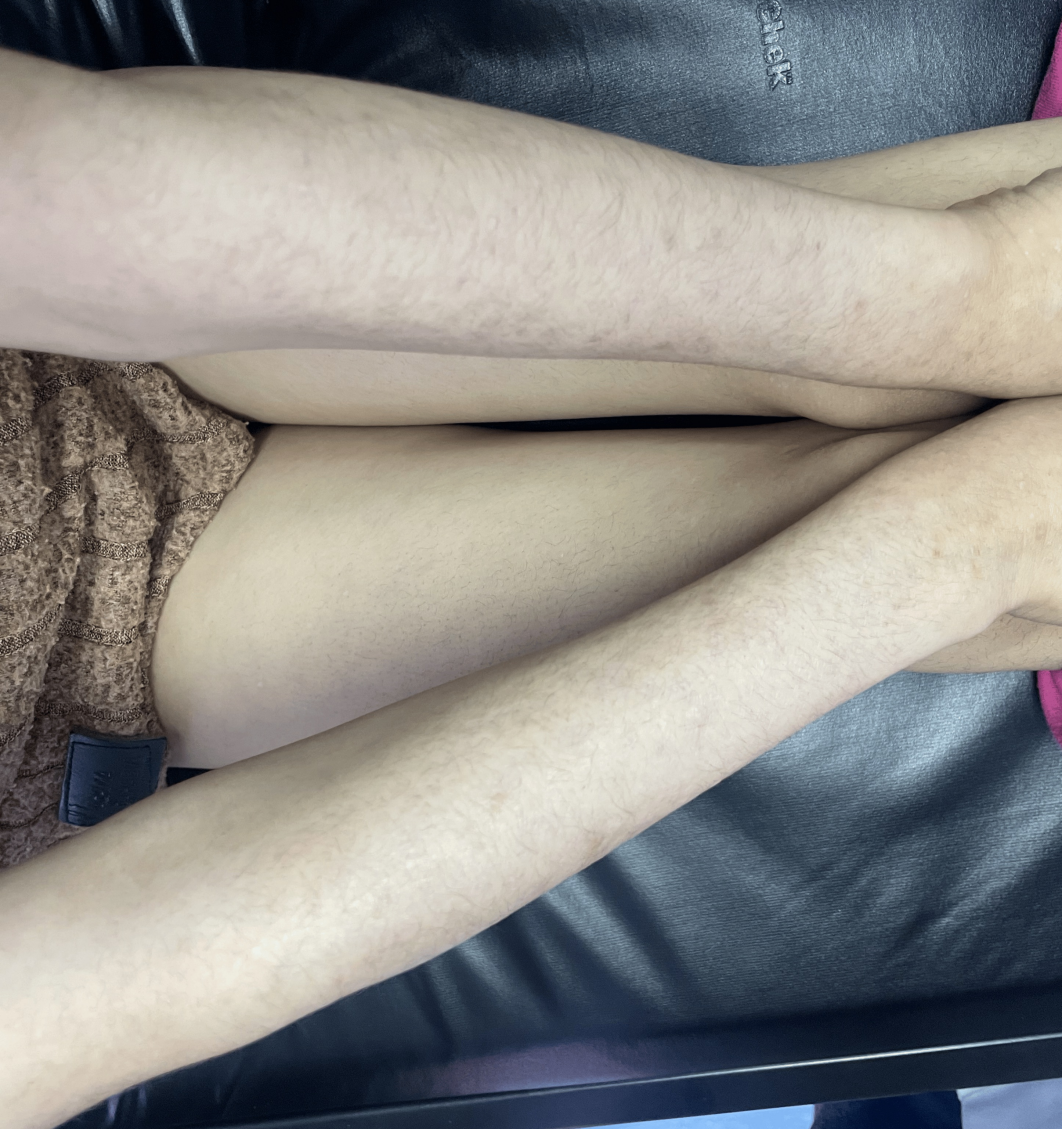
Disappearance of whitish lesions after treatment with corticosteroids. Improvement of the cutaneous lesions.

Biopsies were performed on lesions from both the forearm and the genital region. Histopathological examination of the genital lesion showed epidermal atrophy, vacuolar degeneration of basal keratinocytes, dermal sclerosis, and a mononuclear inflammatory infiltrate. The biopsy from the forearm demonstrated the following similar features: epidermal atrophy, hyperkeratosis, dermal sclerosis, and mononuclear cell infiltration. Based on the clinical presentation, dermoscopic findings, and histopathology, a diagnosis of disseminated lichen sclerosus involving both genital and extragenital sites was confirmed. Treatment with topical clobetasol propionate 0.05% was initiated. After two months, the genital lesions showed significant improvement; however, new extragenital lesions continued to appear. Therefore, a therapeutic trial of intramuscular corticosteroid injections was administered once a month for two months. Follow-up revealed marked regression of the lesions, with no signs of recurrence (Figure 3).

## Discussion

Lichen sclerosus or lichen sclerosus et atrophicus is a chronic immune-mediated disease affecting mucocutaneous areas [1]. Lichen sclerosus can occur at any age, but especially in prepubertal girls and after menopause in women, suggesting a role for low estrogen levels during these periods. The incidence of pediatric LS (ages 0-20 years) in the general population is estimated at 0.04-0.06%, compared with 0.1-0.6% in adults [2]. The pathogenesis remains unclear, but it is multifactorial. Genetic predisposition, autoimmune mechanisms, and environmental influences have all been implicated as contributing factors. Several studies have reported associations between LS and other autoimmune disorders, including alopecia areata and autoimmune thyroid disease [3].

The anogenital region is the most commonly affected site in lichen sclerosus; however, extragenital involvement is rarely reported in children. In girls, the most frequent symptoms include pruritus, burning sensation, and dysuria, while in boys, phimosis and balanitis are the most common presentations. Clinically, lichen sclerosus presents as porcelain-white plaques with an atrophic aspect in extragenital areas, and fissures or erosions in the genital site. These clinical features are highly suggestive of lichen sclerosus; however, differential diagnoses, such as vitiligo and morphea, should be considered [4].

In most cases, clinical examination is sufficient for diagnosis, but histopathological confirmation is recommended when the diagnosis is uncertain. Histology typically reveals epidermal and dermal changes, including hyperkeratosis, epidermal atrophy, basal cell degeneration, and dermal fibrosis. Dermoscopy can be a helpful non-invasive tool, often showing well-demarcated whitish areas with comedo-like openings, which can support the clinical impression [5].

A systematic review of 4516 pediatric lichen sclerosus cases showed that 2.8% of female patients presented with extragenital lesions only, while 2.9% had both extragenital and genital involvement. In contrast, only 0.4% of male patients presented with extragenital lesions [6]. In this study, we report a case of an eight-year-old girl who developed multiple whitish plaques on the legs and forearms bilaterally, two years after the onset of genital lesions. The diagnosis of lichen sclerosus was confirmed through histopathological examination, as differential diagnoses such as lichen planus, psoriasis, vitiligo, and morphea were initially considered. To date, few pediatric cases of lichen sclerosus with extragenital involvement have been reported in the literature.

Management of lichen sclerosus in the pediatric population primarily involves the use of ultrapotent and potent topical corticosteroids due to their anti-inflammatory effects. A few case series have reported good results using topical tacrolimus to treat lichen sclerosus in children [7]. A retrospective analysis of pediatric patients with lichen sclerosus shows that tacrolimus improves signs and symptoms but may be less effective than 0.05% clobetasol propionate (CP) [8].

In the present case, topical corticosteroid treatment led to improvement of the vulvar lesions, but the extragenital lesions remained resistant. As a result, intramuscular corticosteroid injections were administered, with a good clinical response observed within two months. To our knowledge, there are few published reports documenting the use of intramuscular corticosteroids for the treatment of lichen sclerosus in children. However, intramuscular corticosteroids, such as triamcinolone acetonide, have been used in adults for the management of various chronic inflammatory dermatoses.

The prognosis of pediatric lichen sclerosus remains unknown; the majority of affected girls continued to develop signs and symptoms beyond puberty. Maintenance therapy with topical corticosteroids may help reduce the risk of long-term complications [2]. Although lichen sclerosus is a known risk factor for squamous cell carcinoma in adults, there have been no reported cases of this carcinoma arising in pediatric patients [3].

## Conclusions

Lichen sclerosus in children is a chronic inflammatory skin disease that must be identified and treated promptly to avoid long-term consequences such as scarring, deformity, or functional disturbances. Maintaining a strong clinical suspicion and performing a thorough examination are key steps to ensuring an early and accurate diagnosis. Our case highlights that thorough dermatological evaluation, complemented by dermoscopy, is essential for diagnosing lichen sclerosus in children when biopsy is not possible.
